# Developmental Changes in Accommodation Evidenced by an Ultrabiomicroscopy Procedure in Patients of Different Ages

**Published:** 2013

**Authors:** Giovanna Benozzi, Juliana Leiro, Sonia Facal, Cristian Perez, Jorge Benozzi, Betina Orman

**Affiliations:** 1 Centro de Investigación Avanzada para la Presbicia (C.I.A.P.) . Ciudad Autónoma de Buenos Aires, Republic of Argentina.; 2 Pharmacology Unit, School of Dentistry, Buenos Aires University. Ciudad Autónoma de Buenos Aires, Republic of Argentina.

**Keywords:** Accommodation, Ultrabiomicroscopy, Aging

## Abstract

We demonstrate that changes in the behaviour of the contractile ciliary muscle accompanied by augmented rigidity of the lens are the most important aspects in the loss of accommodation. With ultrabiomicroscopy (UBM), we demonstrated that the performance of the ciliary muscle is diminished and accompanied by rigidity of the lens. Both lens thickness and trabecular-ciliary process distance (TCPD) were the parameters that showed major alterations with the loss of accommodation in patients of different ages. The results indicated that the differences between these parameters in farsightedness and nearsightedness in the different groups of patients were positively correlated.

## INTRODUCTION

During the accommodative process, anatomical changes occur that focus objects located at various distances [1]. In this process, the ciliary muscle contracts, the pupil changes its size and eyes converge (active accommodation). The ciliary muscle contracts causing a lens conformational and positional change (passive accommodation). The accommodation amplitude depends on such active and passive changes [2].

Through accommodation, the ciliary muscle moved centripetally and approaches the back of the iris. The zonular fibers are relaxed allowing the lens to adopt a spherical shape and decrease the radius of the curvature of the anterior and posterior capsules [3]. Simultaneously, the pupil size decreases, which eliminates periphery optical aberrations and increases the focus depth.

Ultrasound biomicroscopy (UBM) is an ideal instrument in visualizing posterior chamber structures that are shielded by the iris [4] and are impossible to study by optical coherence tomography [5] or other optical devices [6]. 

**Figure 1:**
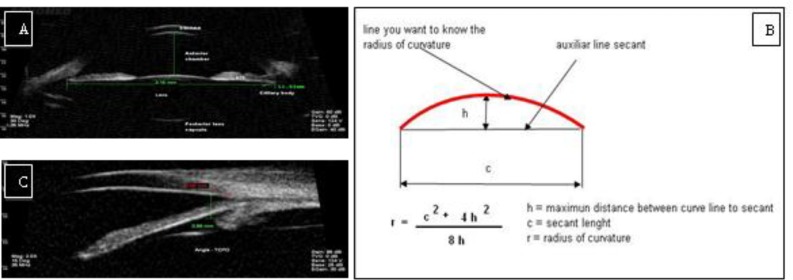
A, C: Images from UBM to obtain measurements of the anterior and posterior chambers of the eye. B, Schematic drawing, demonstrating how to obtain measurements.

**Table 1:** Parameters studied using UBM in different groups of patients

**Conditions**	**Anterior chamber**	**Cornea-posterior capsular lens**	**Lens**	**Radius of curvature AC**	**Radius of curvature PC**	**TCPD**
**Group I**						
**Far (mm)**	2.97 ± 0.10	6.68 ±0.32	3.71 ± 0.08	4.82 ±0.21	2.48 ±0.10	0.81 ± 0.03
**Near (mm)**	2.91 ±0.11	6.80 ±0.33	3.89 ± 0.08	3.18 ±0.15	1.91 ±0.11	0.70 ±0.02
**Group II**						
**Far (mm)**	2.77 ±0.12	6.69 ±0.33	4.03 ±0.09	4.83 ±0.18	2.41±0.10	0.81 ±0.03
**Near (mm)**	2.73 ±0.13	6.79 ±0.34	4.15 ±0.1	3.73 ±0.17	2.15 ±0.10	0.77 ±0.02
**Group III**						
**Far (mm)**	2.24 ±0.11	6.81 ±0.33	4.30 ±0.22	3.86±0.11	2.09 ±0.23	0.81 ±0.03
**Near (mm)**	2.23 ±0.11	6.86 ±0.34	4.32 ±0.22	3.82 ±0.18	2.02 ±0.21	0.80 ±0.03

**Table 2:** Values of the lens and TCPD with UBM between patient groups considering nearsightedness and farsightedness

**Structure**	**Group I**		**Group II**		**Group III**	
	**Variation(%)**	**P value**	**Variation (%)**	**P value**	**Variation(%)**	**P value**
**Lens**	**4.85**	P<0.0001*	**2.98**	0.0113*	**0.46**	0.7444
**TCPD**	**13.58**	P<0.0001*	**4.93**	0.0182*	**1.23**	0.5612

**Figure 2:**
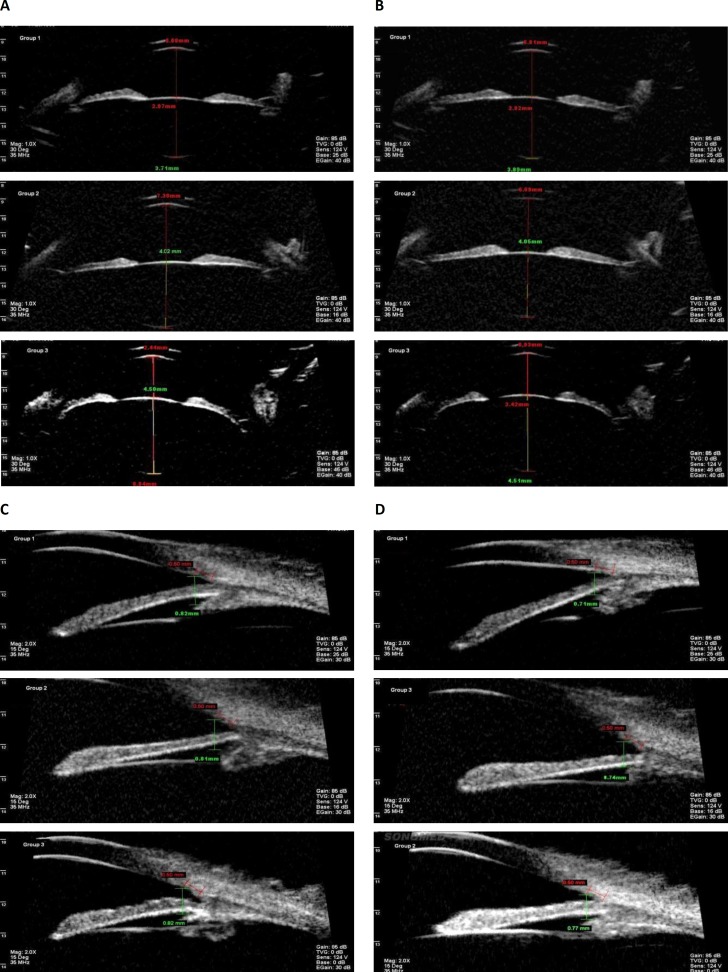
UBM images obtained from the three groups studied. A: Sulcus to sulcus, far. B: Sulcus to sulcus, near. C: Angle, far. D: Angle, near

**Figure 3:**
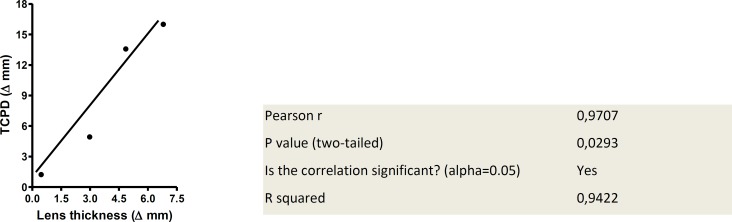
Correlation between Δ Lens thickness and Δ TCPD

Moreover, UBM can be useful for monitoring dynamic changes that occur during accommodation. It can produce images of the relationship between the adjacent intraocular structures, including the crystalline lens, ciliary body, zonule and iris [7]. Its resolution and reproducibility provide the possibility of measuring changes in the distances between these structures. 

To determine the changes during accommodation using this procedure, the images were obtained with the "sulcus to sulcus" program from 9 to 3 o’clock with reference to the pigment epithelium and the posterior lens capsule to obtain centered images. The method used a traced line parallel to the pigment epithelium and a second line orthogonal to the previous one (Figure 1a). This was performed by measuring the radii of the curvature of the anterior and posterior capsules [8,9] of the lens, constructing a secant and determining a trigonometric clearance value, as shown in Figure 1b. For observation of changes at the zonula, ciliary body and ciliary muscle, the "angle" program was used to perform a catch at 12 o’clock. To measure the distance from the corneal endothelium to the anterior surface of the ciliary body, a line was used from the anterior surface of the ciliary body at a point on the corneal endothelium located 500 microns to the scleral spur, as shown in Figure 1c.

## HYPOTHESIS

The human accommodation phenomenon is a dynamic process that alters with age. There is a decrease in the contractile capacity of the ciliary muscle and an augmented rigidity of the lens that takes place during aging, which alters the complex process of accommodation. Our hypothesis was that the loss of accommodation can be estimated by a UBM technique that measures the variations in lens thickness and the trabecular-ciliary process distance (TCPD) in patients of different age groups.

## DISCUSSION

It is known that accommodation decreases gradually from the age of three as much as the structure lens changes [10, 11]. The anterior segment developmental changes that occur from 35 to 65 years old during accommodation can be observed anatomically and biometrically with UBM [12].

We performed a retrospective study on 100 patients that were divided into three groups according to age ranges: group I from 35 to 45 years old (n=36; 20 males and 16 females; median ± SEM, 39±4 y), group II from 46 to 55 years old (n=34; 18 males and 16 females; 49±4 y), and group III from 56 to 65 years old (n=33; 14 males and 19 females; 61±3 y). Only patients that had a refraction of +/- 0.75 diopters and maximum degree binocular fusion participated in the study. No patient in the study had an ocular or systemic disease.

UBM was performed using the Dimension 5150 Vumax (Sonomed, USA) under standard illumination. In order to visualize the posterior lens capsule in all cases, we used a 35 MHz probe [13, 14]. All patients were scanned with the calibrated examiner in the supine decubitus position. A fixation point for the left eye was located on the ceiling approximately 3.0 m away (far) and another was 35 cm away (near). We obtained different pictures of the right eye for near and far accommodation in the same condition [15]. In each, the images were obtained to biometrically measure the anterior chamber (from the corneal endothelium to the anterior lens capsule), the lens (distance between their capsules) and the distance from the cornea to the ciliary body (TCPD) [16]. The large biometric variability found in the population required individual analysis that was objective, dynamic, in vivo and reproducible [17,18,19]. To date, UBM is the most powerful tool available to measure and display biometric and morphological changes, which allows us to assess accommodative functions [12]. 

The UBM images shown here are from patients in groups I, II and III (Figure 2). Experimental results are shown in Table 1, Table 2 and Figure 3, respectively.

The UBM results for the three studied patient groups are shown in Table 1. It can be seen that, in farsightedness, the anterior chamber increased in thickness, whereas in nearsightedness the anterior chamber decreased. The lens diminished its radii of curvature in Groups I and II and there were no changes in group III.

On the other hand, when we studied the thickness of the lens and the TCPD, we obtained significant differences in the studied groups, which are shown in Table 2. We can see that, in groups I, II and III, when lens thickness decreased as a percent of variations, TCPD diminished because the ciliary muscle decreased its contraction significantly.

As increased in age, the differences in lens thickness and the TCPD diminished for both farsightedness and nearsightedness. The patients near and far accommodation abilities were altered. In addition, in Figure 3 we show a positive correlation between lens thickness and TCPD in all groups of patients studied. 

Group I had a large accommodative capacity for both near and far vision. During accommodation for near vision, as the ciliary body moved forward by ciliary muscle contraction, the thickness of the lens increased, the radii of curvature decreased and the lens slid forward, and the size of the anterior chamber decreased. Therefore, in this group, the largest variation in lens thickness was observed. 

In Group II, for distant vision accommodation, the distance from the corneal endothelium to the posterior capsule of the lens and the radii of curvature adopted by the lens for proper accommodation values were similar to those observed in 

Group I. While with accommodation in near vision, the contraction of the ciliary muscle was less, so it decreased the TCPD with respect to group I. The same was observed with variation in lens thickness, reflecting the accommodative difficulty in near vision for this age group. Finally, in Group III, there were no significant differences for accommodation in distant and near vision for all parameters using UBM. It is important to note that accommodation diminished significantly for both nearsightedness and farsightedness in the patients belonging to group III. 

## CONCLUSION

Our findings suggested that the accommodative amplitude diminished during the lifetime for both farsightedness and nearsightedness as evidenced by a decrease in the differences of lens thickness and the TCPD. Indeed, the values for differences in lens thickness correlated with the values for differences in TCPD in patients in groups I, II and III. Moreover, it is likely that in the course of altered accommodation, the capacity of contraction of the ciliary muscle was diminished, first for nearsightedness and later for farsightedness. This study provides new insight into the modulatory role of the ciliary muscle contraction and the developmental changes of the lens that occur to obtain correct accommodation, which were evidenced by UBM.
